# The serendipitous discovery of thoracic malformations in *Lysine ketoglutarate reductase/saccharopine dehydrogenase* mutants of *Drosophila melanogaster*

**DOI:** 10.17912/micropub.biology.002429

**Published:** 2026-10-26

**Authors:** Kevin R. Cook

**Affiliations:** 1 Bloomington Drosophila Stock Center, Department of Biology, Indiana University, Bloomington, IN, United States

## Abstract

A remarkable series of coincidences led the author to show that mutations in a *Drosophila melanogaster* gene encoding a protein responsible for degrading lysine and regulating ecdysone-induced histone methylation are associated with abnormal morphogenesis of the dorsal thorax. Specifically, loss-of-function mutations in the *Lkrsdh* gene, which encodes the Lysine ketoglutarate reductase/saccharopine dehydrogenase protein, result in loss of the scutoscutellar sulcus, the hingelike cuticular groove between the two major divisions of the fly thorax.

**Figure 1. The thoracic phenotypes of Lkrsdh mutants and the characterization of Lkrsdh alleles:** (A) Dorsal view of a wild type ( *Canton-S* ) fly showing the indentation of the dorsal thorax between the scutum and scutellum forming the scutoscutellar sulcus. The normal position of the sulcus is marked by arrowheads in all panels. (B) Dorsal view of an *Lkrsdh* mutant ( *Lkrsdh ^CR70173-TG4.0^ /Df(2L)ED12527* ) showing near absence of the sulcus. There is a smooth transition from the scutum to the scutellum rather than a deep groove. (C) Lateral view of the same fly to demonstrate the rounded, “grooveless” profile. The arrow points to a small clot from hemolymph leakage. As in *grooveless* mutants, leakage in *Lkrsdh* mutants typically occurs in three positions (this position near the humeral bristles, slightly dorsal and posterior to the sternopleural bristles and/or at the lateral ends of the scutoscutellar sulcus), but clots are usually larger than the one depicted here. (D and E) Dorsal views of other individuals with the same genotype showing that the cuticular area where the sulcus would normally form can sometimes appear crumpled and distorted. In flies with this phenotype, the thoracic profiles are still more rounded than the profiles of normal flies. This phenotype also appears in *grooveless* ( *gvl* ) mutants, though it was not mentioned by Cook and Mauthner (2026). F. Newly sequenced *Lkrsdh* alleles. The top lines show the positions of deletions in terms of Release 6 genomic coordinates. In two alleles, extra nucleotides were introduced at the breakpoint junctions by DNA repair processes. For the first four alleles, the bottom lines show N-terminal amino acids remaining from the normal sequences followed by novel residues introduced by frame-shifted translation. For *Lkrsdh ^Δ163^* , the bottom line shows replacement of 61 amino acids of the normal sequence with FFFFFHF. (Note that panel A was recycled from Cook and Mauthner (2026).)

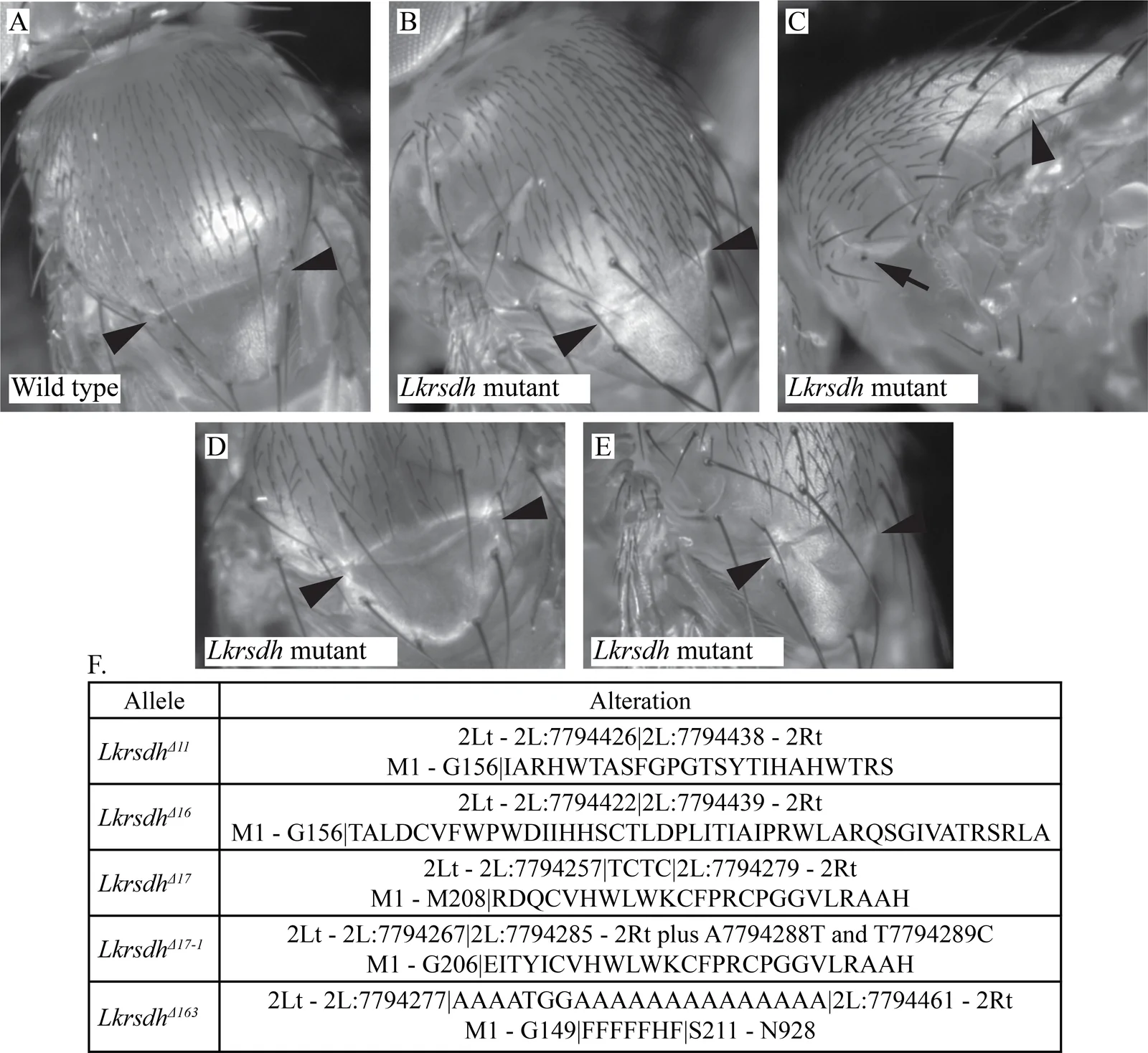



## Description

My colleague Stephanie Mauthner and I recently reported (Cook and Mauthner 2026) that homozygous mutations in the fourth chromosome gene *grooveless* ( *gvl* , formerly *CG31997* ) reduce or eliminate the scutoscutellar sulcus—the transverse cuticular crease separating the larger subdivision of the notum, the scutum, from the smaller subdivision, the scutellum ( Figure 1A ). These mutations also cause hemolymph leakage from the thorax at lower penetrance. The *gvl* gene encodes a protein with a single von Willibrand factor type C (SVWC) domain, likely involved in binding other proteins, and a cell export signal, but no other known protein motifs (Sheldon et al. 2007). SVWC proteins appear to be restricted to arthropods, but the biological functions of few of these proteins have been studied in detail (Labropoulou et al. 2024).

At the same time we were characterizing *gvl* mutations, I was working on an unrelated project involving the *Lkrsdh* gene, which encodes the Lysine ketoglutarate reductase/saccharopine dehydrogenase protein responsible for two enzymatic steps in lysine degradation (Lkr activity in the N-terminal half converts lysine to saccharopine and Sdh activity in the C-terminal half converts saccharopine to alpha-aminoadipic semialdehyde; this bifunctional protein is also called aminoadipate semialdehyde synthase (St. Clair et al. 2017; Tan et al. 2023)). At least in Drosophila, *Lkrsdh* protein also inhibits ecdysone-induced methylation of histone H3 (Cakouros et al. 2008). In an effort to map a mutation that interacts with *Lkrsdh* mutations, I had generated more than a hundred chromosomes carrying a transposon disrupting the *Lkrsdh* gene ( *Mi{ET1}Lkrsdh ^MB01942^* ) linked to a variety of chromosomal deletions, transposon insertions and point mutations located proximal to *Lkrsdh* . When I combined 45 of these “double-mutation” chromosomes in various pairwise combinations in flies via crosses, I was surprised to see the same phenotypes I was seeing in *gvl* mutants: shallow scutoscutellar sulci in crosses with all 45 chromosomes and hemolymph leakage in crosses with 32 of them. To my knowledge, these phenotypes (Figure 1) had never been associated with any gene besides *gvl* and two other genes ( *ascute* and *ascutex* ) identified by Calvin Bridges (Bridges 1935; Bridges and Morgan 1923; Morgan et al. 1925), so it was an amazing coincidence to see them in a different context at the same time I was working with *gvl* mutants!

The causative mutation must have been present on the *Mi{ET1}Lkrsdh ^MB01942^* chromosome used to make the double-mutation chromosomes. For it to have been homozygosed in all the crosses described above, it must lie distal to the *y ^+^* -marked *Mi{MIC}MI04503* transposon insertion in polytene subdivision 30B, because I screened for single crossovers between the *cis* -linked insertions *Mi{ET1}Lkrsdh ^MB01942^* and *Mi{MIC}MI04503* to eliminate *Mi{MIC}MI04503* and place the deletions, insertions and mutations on the same chromosome as *Mi{ET1}Lkrsdh ^MB01942^* . To identify this *gvl* -like mutation, I combined one of the double-mutation chromosomes ( *Mi{ET1}Lkrsdh ^MB01942^ Df(2L)Exel8022* ) with the 54 tiling chromosomal deficiencies from the Bloomington Deficiency Kit that remove 30B to the 2L tip (see Reagents Table). In aggregate, these deficiencies removed 2,983 of the 3,010 genes distal to *Mi{MIC}MI04503* . I found two overlapping deficiencies, *Df(2L)BSC142* and *Df(2L)BSC233,* that failed to complement the mutation. With crosses of the same double-mutation chromosome to an additional seven deficiencies (see Reagents Table), I further narrowed the region with the mutation to the overlap of *Df(2L)ED12527* and *Df(2L)ED499* . Much to my surprise, one of the four genes within this overlap was *Lkrsdh* itself! These deficiencies also failed to complement a sampling of six additional double-mutation chromosomes.

To identify the mutated gene in this small, four-gene region, I complementation tested seven double-mutation chromosomes against mutations in three of the four genes ( *Lkrsdh* , *lncRNA:CR44825* , *Herp* and *r2d2* ) as well as mutations in the genes immediately flanking this region ( *CG7149* and *Cdc14* ) (see Reagents Table). Remarkably, I saw that the only noncomplementing mutation was one in *Lkrsdh* ( *Lkrsdh ^CR70173-TG4.0^* ; Figure 1)! Obviously, the *Mi{ET1}Lkrsdh ^MB01942^* insertion on the double-mutation chromosomes was responsible for the grooveless phenotypes rather than a mutation linked to it.

I have now seen the grooveless phenotypes in complementation crosses with six additional *Lkrsdh* alleles. Four alleles ( *Lkrsdh ^Δ11^* , *Lkrsdh ^Δ16^* , *Lkrsdh ^Δ17^* and *Lkrsdh ^Δ17-1^* ) are CRISPR-induced, frameshift mutations generated in screens by Arthur Luhur and Jason Tennessen ( Figure 1F ). These crosses exhibited phenotypic penetrance and expressivity similar to the crosses described above. These four mutations are positioned to truncate the *Lkrsdh* protein and eliminate domains necessary for both Lkr and Sdh activities as well as the protein region that binds to the ecdysone receptor EcR in regulating histone methylation (Cakouros et al. 2008; Letunic and Bork 2026; Paysan-Lafosse et al. 2025). Lkr activity requires two domains: a catalytic domain and a domain that binds NAD+. The Lkr catalytic domain is likely intact in proteins produced by all four alleles (assuming the truncated proteins are stable), but its activity depends on the domain for binding NAD+, which would be absent—strongly suggesting these four alleles are amorphic. The fifth allele is the original *Lkrsdh* mutation, *Lkrsdh ^1^* , which was previously called *lysine ^1^* (Grell 1961; St. Clair et al. 2017). Interestingly, the scutoscutellar phenotype shows weak penetrance and expressivity in genotypes involving *Lkrsdh ^1^* and hemolymph leakage was rare, suggesting that *Lkrsdh ^1^* is hypomorphic. *Lkrsdh ^1^* is a complex chromosomal rearrangement deleting the normal translation start site, inserting repetitive sequences before the seventh codon and introducing an indel (St. Clair et al. 2017), but my phenotypic results and results presented by St. Clair et al. showing an 80% reduction in transcription suggest there is residual gene expression—likely initiated from a transcription start site within the insertion. The presence of a methionine codon within the inserted sequences near the *Lkrsdh* coding sequences suggests that a variant protein is produced with a shorter, novel N-terminus. The sixth allele ( *Lkrsdh ^Δ163^* ) also appears to be hypomorphic. It came from one of the CRISPR screens mentioned above and consists of 183 bp in the region encoding the Lkr activity replaced by a thymidine-rich sequence (likely TTTTTTTTTTTTTTCCATTTT) that has proved difficult to sequence consistently. The hypomorphic phenotypes suggest the substituted sequence has the appropriate number of thymidines to allow in-frame translation of a variant *Lkrsdh* protein ( Figure 1F ) with a disrupted Lkr domain but intact Sdh and EcR-binding domains.

Flexion between the scutum and scutellum at the scutoscutellar sulcus is part of normal flight (Boettinger and Furshpan 1952; Miyan and Ewing 1985), but, similar to *gvl* mutants (Cook and Mauthner, 2026), I saw that *Lkrsdh* mutants ( *Lkrsdh ^CR70173-TG4.0^ /Df(2L)ED12527* ) with strong grooveless phenotypes are capable of flight. It seems likely that the thoracic malformations in *gvl* and *Lkrsdh* mutants affect flight efficiency, but in neither case has the details of flight mechanics been examined.

I do not know the developmental basis of “groovelessness”. Shallow scutoscutellar sulci and hemolymph leakage could be related to weak or improperly timed flight muscle contractions during pupal development, altered cuticle formation during metamorphosis, or both. Since many biological processes underlie muscle and cuticle development, groovelessness may be a relatively common mutant phenotype. The shallow sulcus phenotype may simply have escaped notice most of the time because it is fairly subtle. (I doubt I would have noticed it in *Lkrsdh* mutants if I had not been working with *gvl* mutants.) Indeed, there may be no close functional relationship between *Lkrsdh* protein and the secreted SVWC protein encoded by *gvl.* Currently, it is impossible to say if the grooveless phenotypes in *Lkrsdh* mutants are attributable to the widespread effects of increased lysine on metabolic processes (St. Clair et al. 2017), to the widespread effects of misregulating ecdysone-dependent processes on development (Morrow and Mirth 2024), or a mixture of both.

## Methods

The stocks used in complementation tests are listed in the Reagents Table. More information about these stocks may be obtained via the website for the Bloomington Drosophila Stock Center (https://bdsc.indiana.edu/). Genetic nomenclature and gene models reflect FlyBase release 2026_03 (Öztürk-Çolak et al. 2024). DNA was extracted from frozen samples of adult flies using standard protocols and sequenced as single-end reads on the Ultima Genomics platform by the University of Minnesota Model Organism Sequencing Service (https://genomics.umn.edu/service/model-organism-sequencing).

## Reagents

**Table:** 

Stock number	Genotype	RRID or source
Deficiency Kit stocks		
90	* Df(2L)C144, dpp ^d-ho^ ed ^1^ /In(2LR)Gla, wg ^Gla-1^ PPO1 ^Bc^ Egfr ^E1^*	RRID:BDSC_90
744	*Df(2L)M24F-B/SM1*	RRID:BDSC_744
3084	*Df(2L)ast2/SM1*	RRID:BDSC_3084
3548	* Df(2L)al, ds ^al^ /In(2L)Cy, Duox ^Cy^*	RRID:BDSC_3548
5330	* Df(2L)ed1/CyO; P{ry ^+t7.2^ =ftz-lacC}1 *	RRID:BDSC_5330
6338	* Df(2L)BSC6, dpy ^ov1^ cn ^1^ /SM6a *	RRID:BDSC_6338
6507	y ^1^ w ^*^ ; Df(2L)drm-P2, P{w ^+mC^ =lacW}ND-PDSW ^k10101^ /SM6b	RRID:BDSC_6507
6648	* Df(2L)dpp ^d14^ /In(2LR)Gla, wg ^Gla-1^*	RRID:BDSC_6648
7144	* Df(2L)BSC37, dpp ^EP2232^ /CyO *	RRID:BDSC_7144
7492	* w ^1118^ ; Df(2L)Exel6005, P{w ^+mC^ =XP-U}Exel6005/CyO *	RRID:BDSC_7492
7495	* w ^1118^ ; Df(2L)Exel6009, P{w ^+mC^ =XP-U}Exel6009/CyO *	RRID:BDSC_7495
7497	* w ^1118^ ; Df(2L)Exel6011, P{w ^+mC^ =XP-U}Exel6011/CyO *	RRID:BDSC_7497
7744	* w ^1118^ ; Df(2L)Exel6277, P{w ^+mC^ =XP-U}Exel6277/CyO *	RRID:BDSC_7744
7783	* w ^1118^ ; Df(2L)Exel7011/CyO *	RRID:BDSC_7783
7807	* w ^1118^ ; Df(2L)Exel7034/CyO *	RRID:BDSC_7807
8673	* w ^1118^ ; Df(2L)BSC107/SM6a *	RRID:BDSC_8673
8674	* w ^1118^ ; Df(2L)BSC109/CyO *	RRID:BDSC_8674
8835	* w ^1118^ ; Df(2L)BSC110/CyO *	RRID:BDSC_8835
8836	* w ^1118^ ; Df(2L)BSC111/CyO *	RRID:BDSC_8836
8901	* w ^1118^ ; Df(2L)ED19, P{w ^+mW.Scer\FRT.hs3^ =3'.RS5+3.3'}ED19/SM6a *	RRID:BDSC_8901
8906	* w ^1118^ ; Df(2L)ED678, P{w ^+mW.Scer\FRT.hs3^ =3'.RS5+3.3'}ED678/SM6a *	RRID:BDSC_8906
8908	* w ^1118^ ; Df(2L)ED94, P{w ^+mW.Scer\FRT.hs3^ =3'.RS5+3.3'}ED94/SM6a *	RRID:BDSC_8908
9176	* w ^1118^ ; Df(2L)ED136, P{w ^+mW.Scer\FRT.hs3^ =3'.RS5+3.3'}ED136/SM6a *	RRID:BDSC_9176
9270	* w ^1118^ ; Df(2L)ED250, P{w ^+mW.Scer\FRT.hs3^ =3'.RS5+3.3'}ED250/In(2L)Cy ^L^ t ^R^ In(2R)Cy, Duox ^Cy^ amos ^Roi-1^*	RRID:BDSC_9270
9341	* w ^1118^ ; Df(2L)ED385, P{w ^+mW.Scer\FRT.hs3^ =3'.RS5+3.3'}ED385/SM6a *	RRID:BDSC_9341
9343	* w ^1118^ ; Df(2L)ED334, P{w ^+mW.Scer\FRT.hs3^ =3'.RS5+3.3'}ED334/SM6a *	RRID:BDSC_9343
9353	* w ^1118^ ; Df(2L)ED5878, P{w ^+mW.Scer\FRT.hs3^ =3'.RS5+3.3'}ED5878/SM6a *	RRID:BDSC_9353
9502	* w ^1118^ ; Df(2L)BSC142/CyO *	RRID:BDSC_9502
9560	* w ^1118^ ; Df(2L)BSC169/CyO *	RRID:BDSC_9560
9600	* w ^1118^ ; Df(2L)BSC165/CyO *	RRID:BDSC_9600
9605	* w ^1118^ ; Df(2L)BSC172/CyO *	RRID:BDSC_9605
9610	* w ^1118^ ; Df(2L)BSC180/CyO *	RRID:BDSC_9610
9615	* w ^1118^ ; Df(2L)BSC188/CyO *	RRID:BDSC_9615
9631	* w ^1118^ ; Df(2L)BSC204/CyO *	RRID:BDSC_9631
9704	* w ^1118^ ; Df(2L)BSC227/CyO *	RRID:BDSC_9704
9708	* w ^1118^ ; Df(2L)BSC233/CyO *	RRID:BDSC_9708
23676	* w ^1118^ ; Df(2L)BSC291/CyO *	RRID:BDSC_23676
23677	* w ^1118^ ; Df(2L)BSC292/CyO *	RRID:BDSC_23677
23680	* w ^1118^ ; Df(2L)BSC295/CyO *	RRID:BDSC_23680
24118	* w ^1118^ ; Df(2L)ED105, P{w ^+mW.Scer\FRT.hs3^ =3'.RS5+3.3'}ED105/SM6a *	RRID:BDSC_24118
24123	* w ^1118^ ; Df(2L)ED247, P{w ^+mW.Scer\FRT.hs3^ =3'.RS5+3.3'}ED247/SM6a *	RRID:BDSC_24123
24124	* w ^1118^ ; Df(2L)ED7853, P{w ^+mW.Scer\FRT.hs3^ =3'.RS5+3.3'}ED7853/SM6a *	RRID:BDSC_24124
24127	* w ^1118^ ; Df(2L)ED489, P{w ^+mW.Scer\FRT.hs3^ =3'.RS5+3.3'}ED489/SM6a *	RRID:BDSC_24127
24132	* w ^1118^ ; Df(2L)ED629, P{w ^+mW.Scer\FRT.hs3^ =3'.RS5+3.3'}ED629/SM6a *	RRID:BDSC_24132
24378	* w ^1118^ ; Df(2L)BSC354/CyO *	RRID:BDSC_24378
24626	* w ^1118^ ; Df(2L)ED50001, P{w ^+mW.Scer\FRT.hs3^ =3'.RS5+3.3'}ED50001/SM6a *	RRID:BDSC_24626
24652	* w ^1118^ ; Df(2L)ED441, P{w ^+mW.Scer\FRT.hs3^ =3'.RS5+3.3'}ED441/SM6a *	RRID:BDSC_24652
24958	* w ^1118^ ; Df(2L)BSC454/SM6a *	RRID:BDSC_24958
24959	* w ^1118^ ; Df(2L)BSC455/SM6a *	RRID:BDSC_24959
25743	* w ^1118^ ; Df(2L)BSC653/SM6a *	RRID:BDSC_25743
26540	* w ^1118^ ; Df(2L)BSC688, P+PBac{w ^+mC^ =XP3.WH3}BSC688/SM6a *	RRID:BDSC_26540
26544	* w ^1118^ ; Df(2L)BSC692, P+PBac{w ^+mC^ =XP3.RB5}BSC692/SM6a *	RRID:BDSC_26544
27381	* w ^1118^ ; Df(2L)BSC810/SM6a *	RRID:BDSC_27381
94697	* w ^1118^ ; Df(2L)ED4651/SM6a *	RRID:BDSC_94697
Additional deficiency stocks		
9274	* w ^1118^ ; Df(2L)ED499, P{w ^+mW.Scer\FRT.hs3^ =3'.RS5+3.3'}ED499/SM6a *	RRID:BDSC_9274
9616	* w ^1118^ ; Df(2L)BSC189/CyO *	RRID:BDSC_9616
9617	* w ^1118^ ; Df(2L)BSC190/CyO *	RRID:BDSC_9617
9618	* w ^1118^ ; Df(2L)BSC191/CyO *	RRID:BDSC_9618
9619	* w ^1118^ ; Df(2L)BSC192/CyO *	RRID:BDSC_9619
24128	* w ^1118^ ; Df(2L)ED501, P{w ^+mW.Scer\FRT.hs3^ =3'.RS5+3.3'}ED501/SM6a *	RRID:BDSC_24128
24129	* w ^1118^ ; Df(2L)ED12527, P{w ^+mW.Scer\FRT.hs3^ =3'.RS5+3.3'}ED12527/SM6a *	RRID:BDSC_24129
Mutations tested in the *Lkrsdh* region		
14288	* y ^1^ w ^67c23^ ; P{y ^+mDint2^ w ^BR.E.BR^ =SUPor-P}CG7149 ^KG06170^*	RRID:BDSC_14288
14997	* y ^1^ w ^67c23^ ; P{y ^+mDint2^ w ^+mC^ =EPgy2}r2d2 ^EY00028^*	RRID:BDSC_14997
16450	* y ^1^ w ^67c23^ ; P{y ^+mDint2^ w ^+mC^ =EPgy2}Cdc14 ^EY10303^*	RRID:BDSC_16450
17058	* w ^1118^ ; P{w ^+mC^ =EP}Herp ^EP2450^*	RRID:BDSC_17058
20640	* y ^1^ w ^67c23^ ; P{y ^+mDint2^ w ^+mC^ =EPgy2}CG7149 ^EY10883^*	RRID:BDSC_20640
29231	* w ^1118^ ; Mi{GFP ^E.3xP3^ =ET1}r2d2 ^MB11618^ /SM6a *	RRID:BDSC_29231
32068	* P{GMR-w.RNAi}13D; P{ry ^+t7.2^ =neoFRT}40A r2d2 ^S165fsX^*	RRID:BDSC_32068
37074	y ^1^ w ^*^ ; Mi{y ^+mDint2^ =MIC}Herp ^MI03683^	RRID:BDSC_37074
54575	y ^1^ w ^*^ ; r2d2 ^1^ P{ry ^+t7.2^ =neoFRT}40A/CyO, amos ^Roi-1^	RRID:BDSC_54575
60537	* y ^1^ w ^67c23^ ; Mi{PT-GFSTF.2}Herp ^MI03683-GFSTF.2^*	RRID:BDSC_60537
79333	y ^1^ w ^*^ ; TI{GFP ^3xP3.cLa^ =CRIMIC.TG4.2}Cdc14 ^CR00871-TG4.2^	RRID:BDSC_79333
92724	y ^1^ w ^*^ ; TI{GFP ^3xP3.cLa^ =CRIMIC.TG4.0}Lkrsdh ^CR70173-TG4.0^ /SM6a	RRID:BDSC_92724
600152	w ^*^ ; Df(2L)LanB1/CyO, P{w ^+mC^ =GAL4-Kr.C}DC3, P{w ^+mC^ =UAS-GFP.S65T}DC7	RRID:BDSC_600152
Additional *Lkrsdh* mutations tested		
692	* Lkrsdh ^1^ rc ^1^ ; ss ^1^*	RRID:BDSC_692
101560	* wg ^Sp-1^ Lkrsdh ^1^ rc ^1^ J ^1^ /SM1 *	Kyoto Stock Center
607941	* w ^1118^ ; Lkrsdh ^Δ11^ /CyO *	RRID:BDSC_607941
	* w ^1118^ ; Lkrsdh ^Δ16^ /CyO *	Jason Tennessen
607942	* w ^1118^ ; Lkrsdh ^Δ17^ /CyO *	RRID:BDSC_607942
	* w ^1118^ ; Lkrsdh ^Δ17-1^ /CyO *	Jason Tennessen
	* w ^1118^ ; Lkrsdh ^Δ163^ /CyO *	Jason Tennessen
Stock used for the photograph of a normal scutoscutellar sulcus		
64349	*Canton-S*	RRID:BDSC_64349
